# Clinical Utility of Intraoperative Tympanomastoidectomy Assessment Using a Surgical Microscope Integrated with an Optical Coherence Tomography

**DOI:** 10.1038/s41598-018-35563-5

**Published:** 2018-11-27

**Authors:** Jaeyul Lee, Ruchire Eranga Wijesinghe, Deokmin Jeon, Pilun Kim, Yun-Hoon Choung, Jeong Hun Jang, Mansik Jeon, Jeehyun Kim

**Affiliations:** 1 School of Electronics Engineering, College of IT Engineering, Kyungpook National University, 80 Daehak-ro, Buk-gu, Daegu 41566 Republic of Korea; 2 Department of Biomedical Engineering, College of Engineering, Kyungil University, 50, Gamasil-gil, Hayang-eup, Gyeongsan-si, Gyeongsangbuk-do 38428 Republic of Korea; 3 Department of Otolaryngology, School of Medicine, Ajou University, 164 World cup-ro, Yeongtong-gu, Suwon, Gyeunggi-do 16499 Republic of Korea

## Abstract

Significant technical and optical advances are required for intraoperative optical coherence tomography (OCT) to be utilized during otological surgeries. Integrating OCT with surgical microscopy makes it possible to evaluate soft tissue in real-time and at a high resolution. Herein, we describe an augmented-reality, intraoperative OCT/microscope system with an extended working distance of 280 mm, providing more space for surgical manipulation than conventional techniques. We initially performed *ex vivo* experiments to evaluate system performance. In addition, we validated the system by performing preliminary clinical assessments of tympanomastoidectomy outcomes in six patients with chronic otitis media. The system evaluated residual inflammation in the region-of-interest of the mastoid bone. Most importantly, the system intraoperatively revealed the connection between the graft and the remnant tympanic membrane. The extended working distance allows otological surgeons to evaluate the status of both the mastoid bone and tympanic membrane during manipulation, affording full intraoperative imaging.

## Introduction

Chronic otitis media (COM) is a common inflammatory disease of the middle ear associated with damage to the tympanic membrane (TM)^1–3^, causing persistent (and sometimes permanent) disability attributable to irreparable middle-ear injury. COM is associated with both otorrhea and large, persistent TM perforations. Mastoid air cell shows inflammatory change according to the progress of disease; COM can be often controlled by either conservative management or minimally invasive, tailored procedure with minimal sequelae. For the cases satisfying the indication for surgery, tympanomastoidectomy is performed to manage COM, eradicating disease by eliminating inflammation and restoring hearing^4,5^. Since the first otorhinolaryngological surgical microscope was introduced in the 20^th^ century, such microscopes have become essential during tympanomastoidectomy^6,7^ Although remarkable progress has been made, surface imaging does not allow visualization of subsurface anatomy. Accurate, noninvasive subsurface data can be beneficial, and their lack renders surgical success crucially dependent on the surgeon’s experience. Thus, optical coherence tomography (OCT) can be well-utilized intraoperatively to afford real-time, high-resolution subsurface (morphological) visualization^8^. OCT yields both *ex vivo* and *in vivo* contrast-free tomographic and volumetric images with axial resolutions of 1–15 μm and sensitivities >110 dB^9,10^. It has found applications in ophthalmology^11^, cardiology^12^, dentistry^13^, and dermatology^14^ and in industrial^15^ and agricultural^16^ settings. Owing to the capability of tissue differentiation and localization of critical structures, several successful middle ear related OCT studies also confirmed the clinical usefulness of OCT in the field of otorhinolaryngology^17–20^. Moreover, the full-field membrane thickness of *in vivo* rabbit and human TM and *ex vivo* human middle ear tissues were visualized by OCT delineating relevant structures and sublayers, such as middle ear ossicles, nerves, and tendons at a higher resolution^21,22^. Due to the ability of visualizing aforementioned structural properties, OCT enables diagnosis of patients with persistent conductive hearing loss providing complementary information helpful for clinical decision^23^. Many groups have integrated OCT with surgical microscopes^24–27^ to improve enface retinal visualization; detect iris incarceration, iridocorneal adhesions that develop during penetrating keratoplasty^28^, and the human thyroid; and quantitatively characterize the efficacy of tympanostomy tube surgery seeking to cure otitis media^29–32^. However, most conventional techniques have small working distances, hindering surgical hand movements; the space between the objective lens of the surgical microscope and the surgical field is small. Both OCT and OCT-photoacoustic tomography (PAT) combined with augmented-reality surgical microscopes have been successfully used by our research group in various *in vivo* animal studies for possible ophthalmological and otorhinolaryngological applications. We employed configurations similar to those of conventional techniques and verified the applicability of our systems^33–35^. We have used OCT to obtain wide-field views of the depth-resolved internal microstructures of diseased TMs and middle ears at high spatial resolution. We have also investigated the depth to which optically cleared cochlear tissues could be visualized, providing fundamental data, and redirecting research interest toward surgical investigations^36–40^.

The present study was motivated by our previous efforts to visualize and diagnose COM using OCT. We used an augmented-reality microscope/OCT system, initially developed to aid ophthalmology surgeons, to facilitate otorhinolaryngological surgery; we extended the working distance to 280 mm via changes in magnification. The enhanced working distance improves the intraoperative visibility and surgical space. Prior to the clinical utility, we applied the developed system to evaluate the clinical applicability by visualizing *ex vivo* TM specimens of guinea pig and mastoids of *ex vivo* human cadaver as a preclinical test. And then, the system was used during tympanomastoidectomy to identify residual mastoid inflammation and the success of TM grafting in six patients. The feasibility was verified through deep imaging and tissue positioning. The obtained augmented-reality and cross-sectional OCT results can be helpful to surgeries enhancing the clinical research. To the best of our knowledge, this constitutes the first clinical use of an augmented-reality surgical microscope/OCT system for intraoperative assessment of tympanomastoidectomy.

## Materials and Methods

All specimens were prepared in line with the guidelines of the Institutional Animal Care and Use Committee of Ajou University (approval no. 2016–0027). The study was approved by the Institutional Review Board of Ajou University Hospital (approval no. AJIRB–DEV–OBS–16–531). All methods employed in this study were in accordance with the approved guidelines and the Declaration of Helsinki. All personal information was kept confidential as required. Informed consent was obtained from all subjects.

### Preparation of *ex vivo* tympanic membranes of guinea pigs and mastoids of human cadavers

A guinea pig (a Hartley albino male, aged 3 weeks, 160–190 g) yielded two TM preparations after intraperitoneal injection of Zoletil 50 (0.1 mL/100 g; Virbac Laboratoire, Korea) and Rumpun 2% (v/v) (0.02 mL/100 g; Bayer, Korea). Both bullae including the intact TMs were carefully extracted. Two cadaveric temporal bones were used to evaluate the mastoid cavity. Soft tissue covering the mastoid cortical bone was removed with a dissector, and then the mastoid cortical bone drilled out using a cutting burr, exposing mastoid air cells. The Körner septum separating the mastoid cavity from the antrum was drilled out and the mastoid cavity was subjected to microscopy/OCT.

### Evaluation of residual mastoid inflammation and TM graft status

Six patients (five males, one female, four right ears, two left ears, age range 20–66 years) with COM were included. Routine preparation and draping were performed under general anesthesia and 1:100,000 (v/v) lidocaine and epinephrine were injected to induce local anesthesia. We created Lempert I, II, and III incisions in the external auditory canal, and elevated a Körner flap. The retroauricular skin was elliptically incised and a periosteal flap was elevated using a microdissector. The retroauricular area was self-retracted to expose the TM, external auditory canal, and mastoid cortical bone. After elevating the tympanomeatal flap, the middle ear cavity was explored and inflammatory tissue was removed. Mastoid cortical bone was removed by burr drilling/cutting and the exposed antrum was also removed. Mastoid mucosal status was evaluated and inflammatory tissue was removed via micro-dissection. If ossicular discontinuity was apparent, ossiculoplasty was performed using either autologous bone or an ossicular replacement prosthesis. The temporalis muscle fascia was prepared for grafting, and was inserted into the tympanum using an underlay technique. The continuity of the remnant TM and the ossicles, and appropriate graft positioning, were evaluated using our system.

### Surgical microscopy/OCT system with extension of the working distance

**Figure 1:**
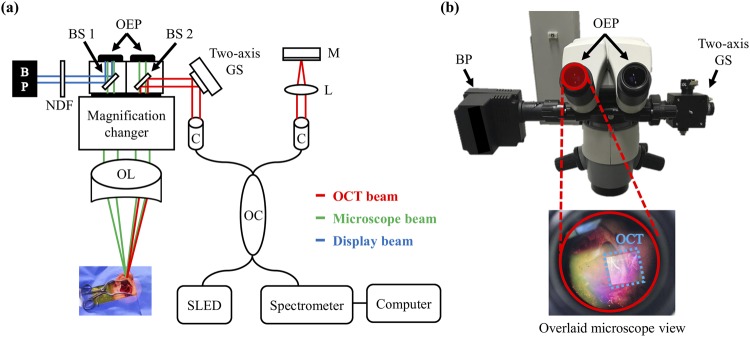
(**a**) Schematic of the surgical microscope/OCT system; (**b**) Surgical head portion of the system. BP: beam projector, BS: beam splitter, OL: objective lens, OEP: ocular eye piece, GS: galvano-scanner, C: collimator, M: mirror, L: lens, OC: optical coupler, NDF: neutral density filter, SLED: super-luminescent diode, OCT: optical coherence tomography.

The augmented-reality microscope/OCT system featuring an extended working distance is schematically illustrated in Fig. 1. Beam splitter (BS) 1 and 2 are important optical components for the augmented implementation as shown in Fig. 1(a). Both beam splitters propagate the surgical microscope beam indicated in green-colored line. The BS 2 simultaneously splits the OCT beam (in red color) towards the objective lens to scan the sample, and the backscattered information transmits towards the spectrometer. The beam projector (BP) shown in the figure is connected to the computer, and the BP beam indicated in blue color projects the OCT image information towards BS 1, where the beam reflects towards the ocular eye piece and displays the OCT image. Thus, the augmented-reality image can be accomplished through the simultaneous visualization of OCT image overlaid on surgical microscope image through the left ocular eye piece as shown in Fig. 1(b). It is worthy to note that the OCT scanning can be visualized through the ocular eye piece, and the position of OCT window can be adjusted or moved according to the surgeon’s convenience using a software based function. The augmented-reality microscope (Huvitz Co., Ltd., South Korea) was linked to a customized spectral-domain OCT (SD–OCT) of central wavelength 846 nm and a bandwidth of 57 nm (SLD–35–HP system; Superlum Ltd., Korea). The in-air OCT resolutions were 8 μm axially and 30 μm laterally. The sensitivity was ca. 102 dB with a near-zero optical delay at an exposure time of 14.1 μs. The details of the SD-OCT system have been published elsewhere^17^. During each imaging session, two-dimensional (500 A-scans) and three-dimensional (500 B-scans) data were obtained at a field-of-view of 10 × 10 × 5 mm.

**Figure 2:**
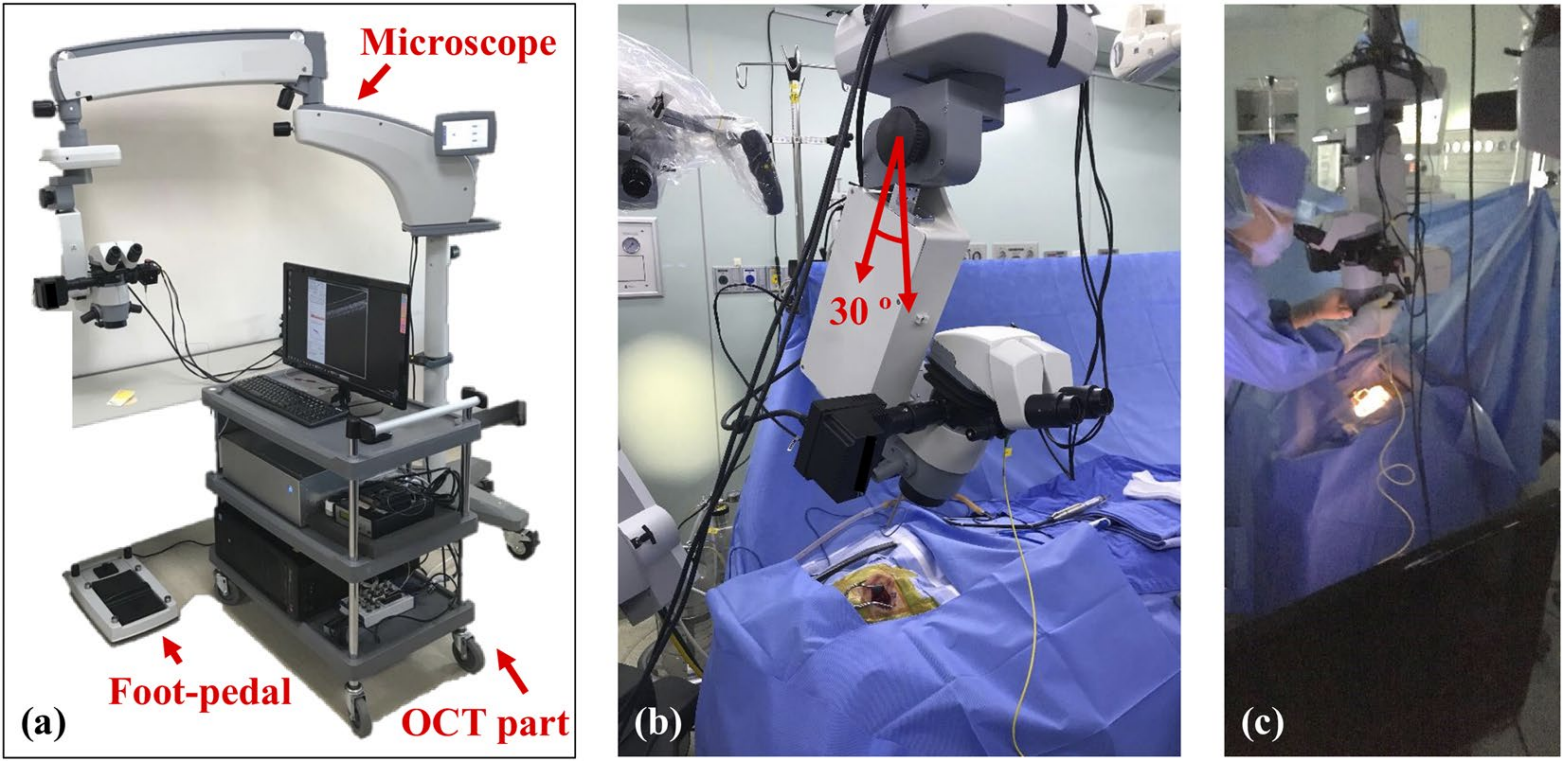
(**a**) Augmented-reality microscope/OCT system; (**b**) the angularly oriented microscope head; (**c**) typical photograph acquisition during surgery.

The augmented-reality microscope featured an eyepiece, a commercially available magnification changer (developed by Huvitz Co., Ltd., South Korea) containing multiple magnification optical lens configuration, an augmented-reality display, and an adjustable (0° or 30°) microscope head. We included a foot pedal to allow the surgeon to use the system; no assistant was required. The OCT working distance was extended to 280 mm using an optical magnification device to create space for surgical manipulation. The adjustable working distance is primarily cooperated by the aforementioned magnification changer. The overall magnification device features an objective lens of focal length 225 mm, a tube lens of effective focal length 160 mm, and several zoom lenses with numerical apertures of 0.015. The total magnification afforded was 10×, associated with a 5% magnification error and 3% distortion. The system is shown in Fig. 2. The surgeon can adjust the head angle, facilitating surgical convenience. The OCT of the developed system was used for the parallel visualization of the surgery along with surgical microscope to provide precise real-time cross-sectional evaluation and confirmation of the surgical region. The surgical microscope of the system was used as the key feature to visualize the entire surgical procedure including residual of mastoid inflammation and TM grafting. More specifically, we used the switching feature (on and off function) to obtain a switched visualization between OCT and surgical microscope, which provided a convenient surgical platform to the surgeon. Additionally, our microscope/OCT system was used to visualize residual mastoid air cell inflammation. Moreover, the foot-pedal control the scanning head position of lateral and axial directions as shown in Fig. 2(a). The Fig. 2(b) shows the manipulation of microscope head angle (0 to 30 degree). Also, we can change the positioning of scanning beam and switching on-off of OCT image on eyepiece. After performing the mastoidectomy and inflammatory tissue eliminations, the microscope/OCT gives a structural depth information of tissues using a cross-section OCT image on the microscope view. It can support to the investigation of the tissues status as shown in Fig. 2(c).

### Software-based GPU-accelerated data processing

**Figure 3:**
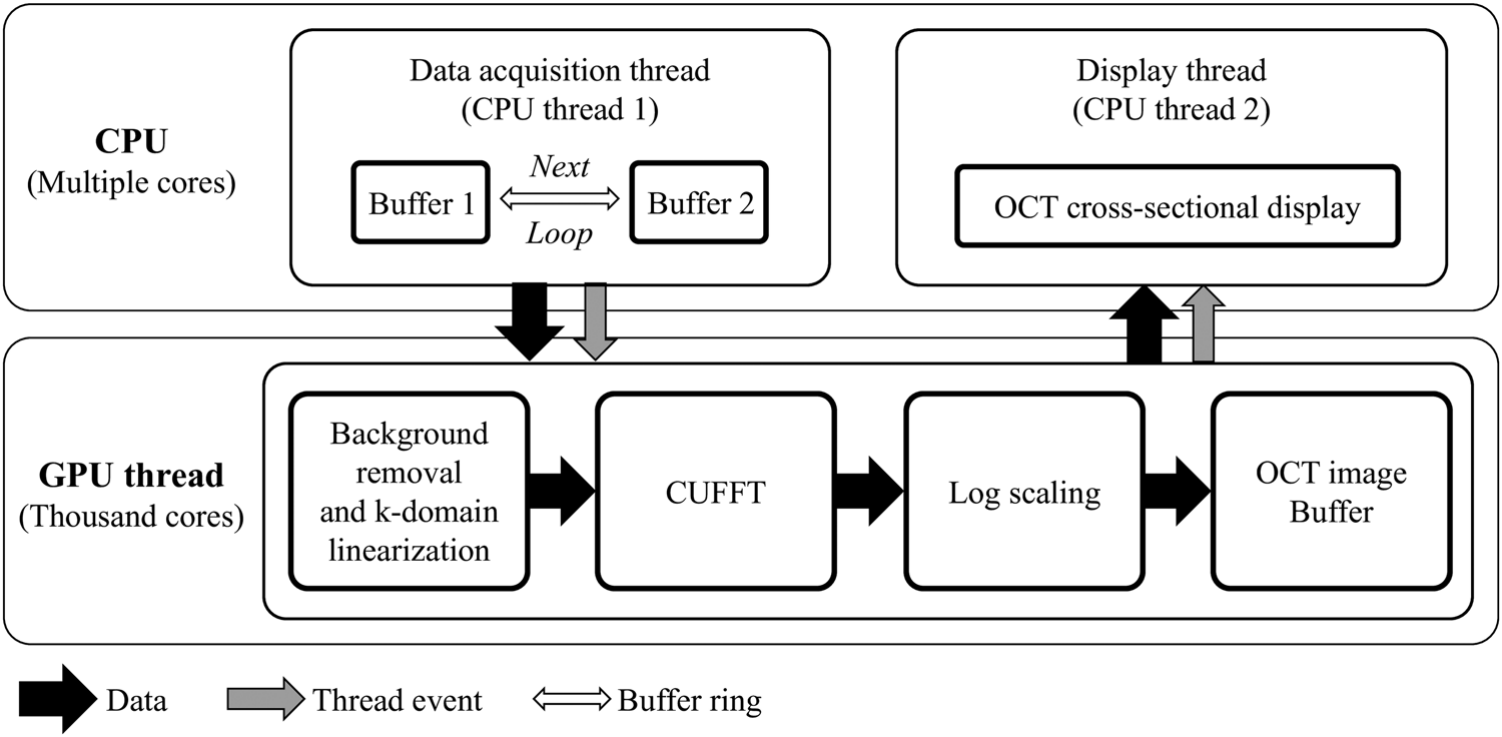
CUDA image processing of the surgical microscope/OCT system.

We used an algorithm to enhance the data-processing speed, enabling real-time, *in vivo* intraoperative OCT imaging. All resampling was implemented using a commercial graphics processing unit (GPU); the required data calculations were performed in multiple parallel streams. The GPU flow chart is shown in Fig. 3, emphasizing the data flow path, thread events, and the buffer ring^41^. The data acquisition thread stored two-dimensional raw signals in the first buffer, allocated them to host memory, and called the signal processing thread. Then the self-iterated acquisition thread repetitively transferred the raw signals to the second buffer without any temporal delay. The signal processing thread copied the frame data stored in the memory buffers. Then full-range wavenumber domain linearization was completed^42^ and the OCT images were transferred back to the host memory and displayed. Thus, the OCT frame rate was immediately enhanced.

## Results and Discussion

### *Ex vivo* tympanic membranes of guinea pigs

**Figure 4:**
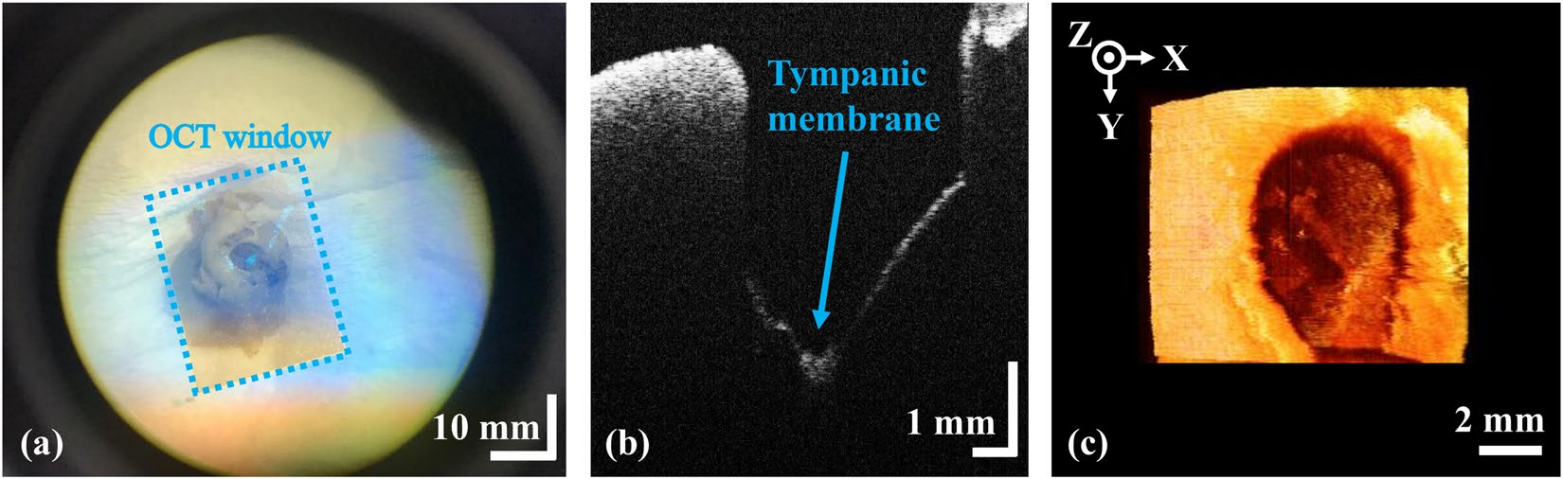
Noninvasive characterization of an *ex vivo* guinea pig TM specimen. (**a**) An overlaid microscopic image; (**b**) a cross-sectional OCT image; (**c**) a volumetric OCT representation.

To confirm the clinical applicability of our system, we noninvasively characterized *ex vivo* TM specimens from guinea pigs prior to clinical assessment. Figure 4 shows the microscopic, cross-sectional, and volumetric OCT images of the TM, respectively. The extended working distance allowed visualization of the deep TM (Fig. 4b). Although we used several lenses to extend the working distance, the bone/soft tissue connection in the region-of-interest was resolved at the micrometer scale, and the morphological properties of the TM were clearly evident. Volumetric reconstruction of the *ex vivo* TM is shown in panel (Fig. 4c). The overlaid microscopic view shows the real-time cross-sectional images of three-dimensional scanning in Supplementary video. The complete volume consisted of 500 images spanning 10 × 10 × 5 mm. The three dimensional data were rendered using commercial software (Avizo, Thermo Fisher Scientific, USA).

### *Ex vivo* imaging of a mastoid bone from a human cadaver

**Figure 5:**
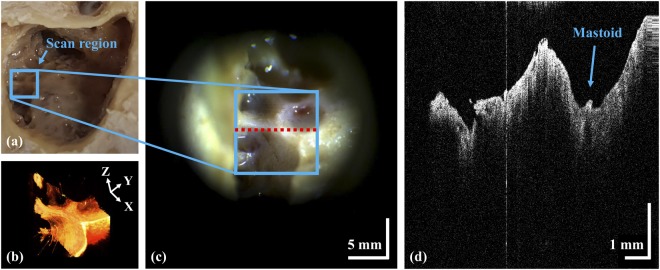
(**a**) The mastoid region of a cadaver; (**b**) volumetric OCT image; (**c**) surgical microscopic view; (**d**) cross-sectional OCT image.

Figure 5 shows a graphical representation of an *ex vivo* mastoid bone from a human cadaver including surgical microscopic, cross-sectional, and volumetric images. We examined the region-of-interest imaging for the cadaver sample before the clinical trials, and checked whether the scanned region could be matched accurately with the eyepiece view on the microscope. As shown in Fig. 5(a,c), surgical microscope information is limited to surface information. Unlike surgical microscope, OCT provides sub-surface anatomy with approximate depth information of the desired region-of-interest at various planes as shown in representative Fig. 5(d). Moreover, non-destructively rendered volumetric information further confirm the potential merits of OCT over surgical microscope as shown in Fig. 5(b). OCT yielded a clear image of the bony surface of the cells. Thus, the system could be used during tympanomastoidectomy. Inflammation thickens the mucosa of mastoid air cells in COM patients. Using a surgical microscope, most inflamed tissue can be visualized and removed. However, remnant inflammation remains and may cause otitis media recurrence. Using our system, the mastoid region-of-interest can be completely evaluated during surgery.

### Intraoperative assessment of residual mastoid inflammation

**Figure 6:**
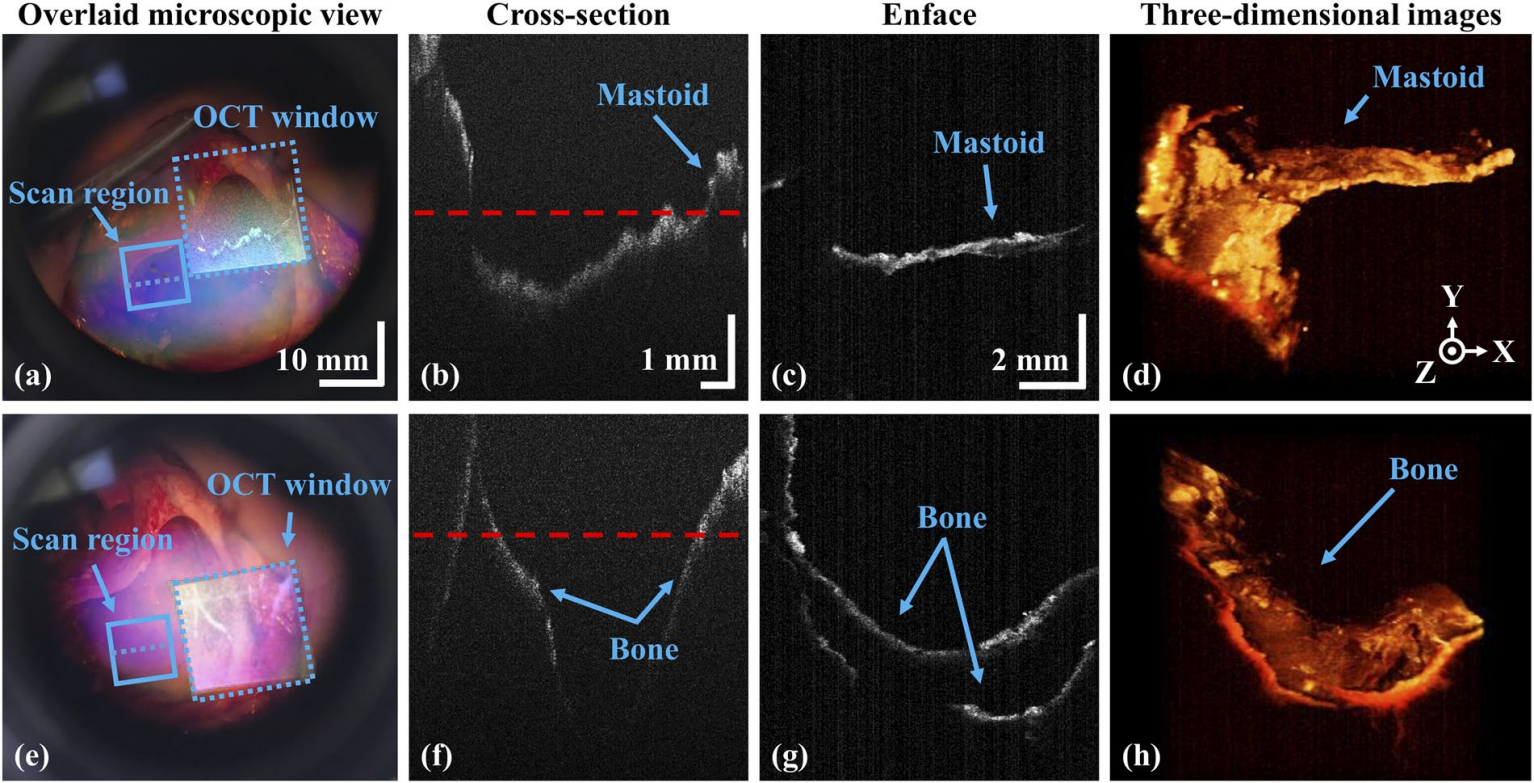
Clinical assessment of *in vivo* human residual mastoid inflammation. (**a**) and (**e**) Microscopic views; (**b**) and (**f**) cross-sectional OCT images; (**c**) and (**g**) the corresponding enface OCT representations along the red dashed lines indicated on the cross-sectional images; (**d**) and (**h**) volumetric OCT images.

Tympanomastoidectomy was performed on six COM patients, and the developed system was well-utilized to evaluate remnant inflammation of the mastoid surface (Fig. 6). The augmented-reality OCT window (indicated in blue color dotted square region) guided the surgeon, providing robust information on the progress of inflammation removal. We similarly evaluated the second surgical region lying underneath the first region; this was possible because of the extended working distance. And the status of inflammatory removal was precisely monitored by using the cross-sectional observations in real-time; which was difficult using the microscope alone.

### Assessment of continuity between the grafted and remnant TM

**Figure 7:**
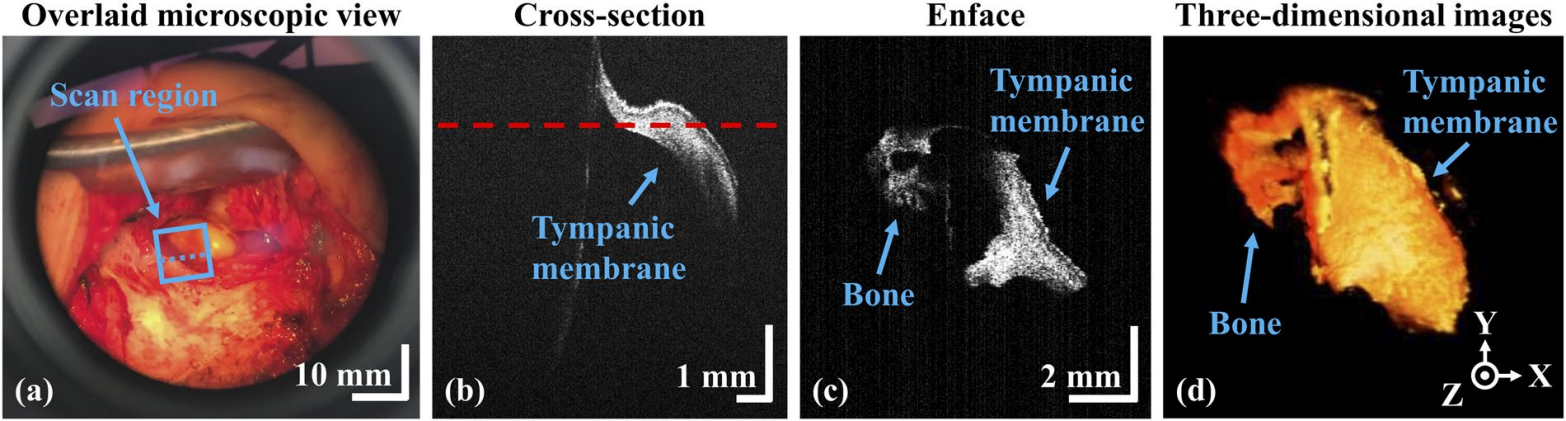
Clinical assessment of a grafted tympanic membrane. (**a**) Microscopic view (OCT window off); (**b**) cross-sectional OCT image; (**c**) enface OCT representation; (**d**) volumetric OCT image.

Each TM was reconstructed using temporalis muscle fascia inserted under the remnant TM after the middle ear cavity was filled with absorbable spongy material. The graft overlapped the perforation margin to prevent later re-perforation. Grafting was intraoperatively assessed using our system. The intraoperative OCT system could offer the information of TM graft connecting and localized tissue positioning for the evaluations. As shown in Fig. 7, a deeper TM structure was observed at the extended working distance of 280 mm confirming the successful grafting approach. Furthermore, the volumetric image yields noninvasive three-dimensional information. All six grafts were evaluated in the outpatient department 14 days after surgery; all were well-adapted.

**Figure 8:**
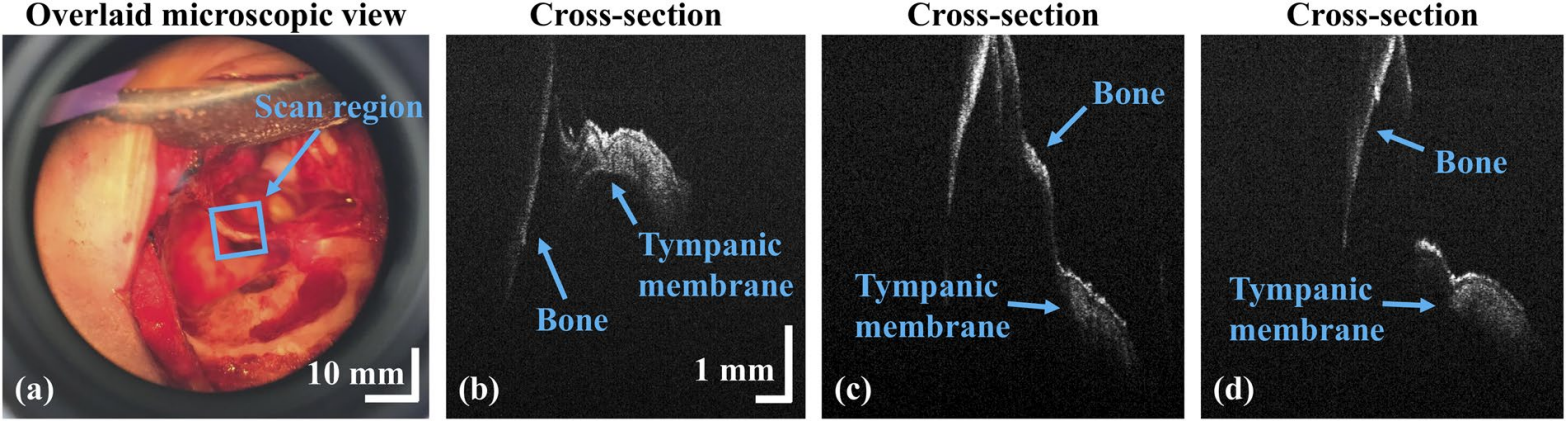
Assessment of the tympanic membrane during surgery. (**a**) Microscopic view; (**b**) to (**d**) cross-sectional OCT images acquired at different positions.

Figure 8 shows another region of the grafted TM connected to bony structure, and the orientation with respect to the patient, showcasing the capacity of our system. The microscope reveals the connection between the external auditory canal and the grafted TM region. The cross-sectional representation yields clear morphological data and the thickness of the soft bony region to which the TM is connected. Our *ex vivo* experiments provided a robust platform for *in vivo* assessments.

The microscopic images of Figs 6(a,e), 7(a) and 8(a) were captured using a video camera to illustrate the augmented-reality visualization of the system. The frame rate (frames per seconds; fps) mismatch between OCT window and the video camera and the slight mismatch of focus between optical lenses of video camera and ocular eye piece are the main causes for the low resolution of Fig. 6(a,e), while the absence of OCT window resolves the negative impact of the image as shown in Figs 7(a) and 8(a). Later, it was confirmed that this limitation was not existed when the dual ocular eye pieces were viewed through naked eyes. Moreover, the acquired cross-sectional and enface representations were well-utilized for the evaluation of inflammation removal and successful TM grafting. The complete removal of inflammation as well as successful TM grafting were performed and confirmed by expert surgeons. Hence, the success of surgery was verified by expertise through the observations of surgical microscopic-OCT visualizations and according to the existing standard surgical reports^5,43,44^. One of the main expectations of the otorhinolaryngological surgeon was to use the developed surgical microscope integrated OCT system as a qualitative confirmation tool to verify the success of the tympanomastoidectomy during the removal of inflammation and grafting. Quantifications of this trend^21^ including the analysis of TM thickness variation through a monitoring process could provide further insight into fundamental anatomy of the surgical region enabling to understand more precise and representative distribution. The aforementioned quantifications will be studied in the next step. In addition, the hearing status and TM appearance were evaluated to confirm postoperative recovery at three weeks after the tympanomastoidectomy. In the future works, we can anticipate to yield significant results when the changes of postoperative TM thicknesses and otoscope images are diversely analyzed with functional audiometry and tympanometry.

## Conclusion

We used an augmented-reality surgical microscope/OCT system during tympanomastoidectomy to evaluate residual mastoid inflammation and TM reconstruction in real-time. The magnification changes afforded by the microscope allows the working distance to range from 250–280 mm, greater than that of existing surgical OCT systems. *Ex vivo* specimens, including TMs from guinea pigs and mastoid bones from human cadavers, were examined prior to any clinical application. Tympanomastoidectomies were performed in six patients with COM; we performed intraoperative imaging on 25 occasions. During tympanomastoidectomy, elimination of gross inflammation/residual mastoid inflammation was noninvasively assessed to confirm surgical success. Similarly, grafting during TM reconstruction was examined. The cross-sectional, enface, and volumetric representations yielded detailed data on the aforementioned microstructures, confirming successful tympanomastoidectomy. Thus, our system can be used to perform tympanomastoidectomy, and will find other real-time applications in otorhinolaryngology. The extended working distance enables the surgeon to operate conveniently. Therefore, we believe that our system will contribute significantly to improvements in otorhinolaryngology.

## Electronic supplementary material

Supplementary Video
